# Household Income Relationship With Health Services Utilization and Healthcare Expenditures in People Aged 75 Years or Older in Japan: A Population-Based Study Using Medical and Long-term Care Insurance Claims Data

**DOI:** 10.2188/jea.JE20180055

**Published:** 2019-10-05

**Authors:** Shota Hamada, Hideto Takahashi, Nobuo Sakata, Boyoung Jeon, Takahiro Mori, Katsuya Iijima, Satoru Yoshie, Tatsuro Ishizaki, Nanako Tamiya

**Affiliations:** 1 Research Department, Institute for Health Economics and Policy, Association for Health Economics Research and Social Insurance and Welfare, Tokyo, Japan; 2 Department of Health Services Research, Faculty of Medicine, University of Tsukuba, Ibaraki, Japan; 3 National Institute of Public Health, Saitama, Japan; 4 Division of Health Service for the Disabled, National Rehabilitation Center, Seoul, Republic of Korea; 5 Institute of Gerontology, The University of Tokyo, Tokyo, Japan; 6 Department of Health Policy and Management, School of Medicine, Keio University, Tokyo, Japan; 7 Tokyo Metropolitan Geriatric Hospital and Institute of Gerontology, Tokyo, Japan

**Keywords:** aged, income, inequality, inequity, socioeconomic status

## Abstract

**Background:**

This study aimed to determine whether there are disparities in healthcare services utilization according to household income among people aged 75 years or older in Japan.

**Methods:**

We used data on medical and long-term care (LTC) insurance claims and on LTC insurance premiums and needs levels for people aged 75 years or older in a suburban city. Data on people receiving public welfare were not available. Participants were categorized according to household income level using LTC insurance premiums data. The associations of low income with physician visit frequency, length of hospital stay (LOS), and medical and LTC expenditures were evaluated and adjusted for 5-year age groups and LTC needs level.

**Results:**

The study analyzed 12,852 men and 18,020 women, among which 13.3% and 41.5%, respectively, were categorized as low income. Participants with low income for both genders were more likely to be functionally dependent. In the adjusted analyses, lower income was associated with fewer physician visits (incidence rate ratio [IRR] 0.90; 95% confidence interval [CI], 0.87–0.92 for men and IRR 0.97; 95% CI, 0.95–0.99 for women), longer LOS (IRR 1.98; 95% CI, 1.54–2.56 and IRR 1.42; 95% CI, 1.20–1.67, respectively), and higher total expenditures (exp(β) 1.09; 95% CI, 1.01–1.18 and exp(β) 1.09; 95% CI, 1.05–1.14, respectively).

**Conclusions:**

This study suggests that older people with lower income had fewer consultations with physicians but an increased use of inpatient services. The income categorization used in this study may be an appropriate proxy of socioeconomic status.

## INTRODUCTION

Japan is facing one of the most rapid aging processes of its population in the world. The number of people aged 75 years or older is projected to increase markedly and reach nearly one-fifth of the population by 2025. Moreover, the increase in life expectancy is higher than that of disability-free or “healthy” life expectancy worldwide, including in Japan,^1^^,^^2^ suggesting an increased need for not only medical services but long-term care (LTC) services as well. These estimations imply health services use and the associated expenditures for this population will impose a large burden on the Japanese society and healthcare systems in the near future.

Public insurance systems for medical and LTC services have been separately implemented in Japan. All people who reach 75 years of age, except for those receiving public assistance, were transferred to the Late Elders’ Health Insurance scheme, which has been managed by local governments since 2008.^3^ The LTC insurance system was introduced in 2000 to help older people lead more independent lives and reduce the burdens of family carers.^4^ The co-payment rates for medical and LTC services are generally 10% for people aged 75 years or older and high out-of-pocket payments above thresholds based on income are capped. Due to the separate implementation of medical and LTC insurance systems and unavailability of personal identification numbers in healthcare, a limited number of studies had hitherto evaluated medical and LTC services together.^5^^,^^6^

Socioeconomic status is an important determinant of health. Several studies have shown that a lower socioeconomic position is typically associated with a negative health status in older people.^7^ There are also studies demonstrating that the impact of socioeconomic status on health decreases or diminishes as people age.^8^ As poverty or lower income is one of the most important components or examples of lower socioeconomic status, people with low income have higher mortality,^9^^–^^11^ morbidity and disability,^8^^,^^12^^,^^13^ and worse patient-reported outcomes.^9^^,^^14^

Specifically, poorer health in people with low income may be caused by limited healthcare access compared with their counterparts, which can be evaluated using health services utilization.^15^^–^^21^ Previous studies suggested people with lower incomes were less likely to use services with high co-payment rates, including dental care and prescription medicines,^15^ and to visit physicians.^16^^,^^17^ Some studies on Japan have shown financial and non-financial barriers to healthcare in older people^18^ and poorer access to outpatient care and more serious health conditions in people with lower income.^19^ Inequalities in access to outpatient and inpatient services due to income were identified in adolescents and the middle-aged after adjustment for their medical needs.^20^ It has been one of the major policy objectives to reduce inequalities in health status and to achieve adequate access to healthcare based on the needs of the Organisation of Economic Co-operation and Development (OECD) countries.^17^ However, there are still limited data available on inequalities in health or access to healthcare for older people in Japan. We thus aimed to determine whether there are disparities in health services utilization in people aged 75 years or older according to household income under the current healthcare system in Japan.

## MATERIALS AND METHODS

### Data source

We obtained data on medical and LTC claims submitted in a middle-sized suburban city in the Tokyo metropolitan area in Japan between April 2012 and September 2013 (∼400,000 residents; ∼8.7% were aged 75 or older as of October 2012) with permission from the local government. We also used data on LTC insurance premiums and certificates of LTC needs levels in terms of required LTC.^6^^,^^21^ All data were anonymized, with unique numbers assigned to each insured person to allow for identification across medical and LTC insurance claims data before we received the data. During the anonymization process, the birth year was approximated by converting into a 5-year range; for example, people born between 1930 and 1934 were between 77 to 81 years of age as of January 1, 2012. We did not receive claims data on medical services for individuals covered by public assistance and consequently did not include them in the analysis.

### Household income

Despite the importance of socioeconomic information for health services research, this information is typically not available from routinely collected medical data. We used data on LTC insurance premiums, which was used in a previous study,^22^ to classify participants according to household income. People aged over 40 years make mandatory payments of LTC insurance premiums based on household income. In this study, participants were categorized into the low-income group if their premium category was equivalent to a level where they and all their family members were exempted from resident taxation, with the remaining being categorized as the middle-to-high income group. This threshold is often used for policy measures to benefit the people who are financially disadvantaged.

### Participants

The participant selection is shown in eFigure 1. All beneficiaries of the Late Elders’ Health Insurance were first selected as potential study population (*N* = 38,876). We limited the data to participants aged 75 years or older by excluding the younger people with disabilities who were eligible for the Late Elders’ Health Insurance system (*N* = 38,379). However, because of the data availability for 5-year ranges of birth years, we could not completely exclude people below 75 with disabilities and eligible for the Late Elders’ Health Insurance system. Subsequently, we selected participants with data on LTC insurance premiums at the baseline (*N* = 37,922), where participants aged 75 years or older who contributed to the database for at least 12 months were included in the analyses (*N* = 30,872). The cohort entry for each participant was defined as the first month when any type of claim was issued. Thus, participants who did not use any medical or LTC services were not included in this study. The registration of individuals within the database was ensured by at least one type of claims data issued in or after the 12 months from the cohort entry. This study was approved by the Ethics Committee at University of Tsukuba (No. 1184).

### Measurements

The LTC needs levels at cohort entry were determined as either support 1 or 2 and care 1 to 5 (most dependent) based on the certificate data for LTC needs levels.^6^^,^^21^ Generally, individuals with support levels receive assistance for daily living and preventive LTC services, and those with care levels receive more extensive LTC services, including in-home, community-based, and facility services. A nationally standardized certification process is applied to determine eligibility and LTC needs levels with computer-aided assessment based on interviews, primary physicians’ opinions, and the subsequent review by the Care Needs Certification Board. In this process, individual’s physical and cognitive functions are assessed, but their income levels are not considered. Participants categorized as “independent” included those who had not applied for certifying their LTC needs and those not certified. Medical services utilization was evaluated in terms of frequency of physician visits and a length of hospital stay (LOS). Physician visits included physicians’ home visits on a regular basis, which were provided for people who could not visit physician’s office or hospitals due to their poor health status. We excluded some surgical procedures (eg, for cataracts or hernia) from LOS evaluation because the LOS was unclear for these services due to the episode-based bundled payment applied, while the medical expenditures associated with these surgical procedures were included in those for inpatient services. Healthcare expenditures were calculated as total and per medical, LTC, and individual health services, including inpatient, outpatient, pharmaceuticals and pharmacy services by community pharmacy, institutional care, and home care services. We classified LTC services into institutional care services, including three designated types of LTC facilities and other types of facilities or group homes for older people. Data on home-visit nursing services under medical insurance and for dental care were not available for this study.

### Analysis

We analyzed data on medical services utilization and healthcare expenditures for the first 12 months from the cohort entry. Descriptive statistics were used to summarize data for men and women separately because of interactions observed for medical services use between the household income category and sex (*P* = 0.001 for physician visits; and *P* = 0.030 for LOS). Since the majority of the study population had not used inpatient and LTC services, summary statistics were also calculated for users of these services during the 12-month study period. Negative binomial regression was used to evaluate the relationship of low income with physician visit frequencies and LOS. Generalized linear models with a gamma distribution and log link function were then used to evaluate the association of low income with healthcare expenditures. All analyses were adjusted by 5-year age group (treated as a categorical variable; 72–76, 77–81, 82–86, 87–91, and ≥92 years) and LTC needs level as a proxy for general health status (treated as a categorical variable; independent, support 1 and 2, and care 1 to 5). Analyses were repeated in dependent or independent participants separately. Expenditures are mostly presented in Japanese yen (JPY), equivalent to 104 United States dollars (USD) according to the OECD purchasing power parities for GDP in 2012. All analyses were conducted using Stata version 14 (Stata Corp., College Station, TX, USA).

## RESULTS

### Characteristics of study population

The study included 12,852 men and 18,020 women. The proportions of participants categorized as low income were highly gender dependent: 1,704 men (13.3%) and 7,483 women (41.5%) (Table 1). One-sixth to 40% of the participants were dependent across groups by gender and household income category, with women and participants with lower incomes being more likely to be functionally dependent. Baseline characteristics for sub-populations who used inpatient, institutional, or home services are shown in eTable 1, eTable 2, and eTable 3.

**Table 1.** Baseline characteristics of study population

		Men		Women	
	
		Low income (*N* = 1,704)	Middle-to-high income (*N* = 11,148)	Low income (*N* = 7,483)	Middle-to-high income (*N* = 10,537)
Age group, years	72–76^a^	614 (36.0)	3,747 (33.6)	1,628 (21.8)	3,219 (30.1)
	77–81	691 (40.1)	4,368 (39.2)	2,409 (32.2)	3,831 (36.4)
	82–86	280 (16.4)	2,139 (19.2)	1,793 (24.0)	2,158 (20.5)
	87–91	95 (5.6)	719 (6.5)	1,104 (14.8)	958 (9.1)
	≥92	24 (1.4)	175 (1.6)	549 (7.3)	371 (3.5)

LTC needs level	Independent	1,246 (73.1)	9,287 (83.3)	4,437 (59.3)	7,825 (74.3)
	Support 1	47 (2.8)	283 (2.5)	386 (5.2)	413 (3.9)
	Support 2	43 (2.5)	220 (2.0)	389 (5.2)	346 (3.3)
	Care 1	82 (4.8)	402 (3.6)	582 (7.8)	612 (5.8)
	Care 2	111 (6.5)	406 (3.6)	576 (7.7)	547 (5.2)
	Care 3	74 (4.3)	272 (2.4)	434 (5.8)	360 (3.4)
	Care 4	62 (3.6)	176 (1.6)	335 (4.5)	221 (2.1)
	Care 5	39 (2.3)	102 (0.9)	344 (4.6)	213 (2.0)

LTC, long-term care.

Figures are shown as frequencies (%).

^a^People <75 were only included if eligible for the Late Elders’ Health Insurance system.

### Medical services utilization

Descriptive statistics on annual medical services utilization are summarized in Table 2, and the relationships of low income with physician visits and LOS are shown in Table 3. Physician visits were infrequent for men (mean 14.1 vs 16.1 days per year; *P* < 0.001) and women (14.8 vs 16.1; *P* < 0.001) with lower income. In the adjusted analyses, low income was associated with fewer physician visits for both men (incidence rate ratio [IRR] 0.90; 95% confidence interval [CI], 0.87–0.92; *P* < 0.001) and women (IRR 0.97; 95% CI, 0.95–0.99; *P* = 0.001). Inpatient services use was more frequent for men than women. LOS was longer for men (mean 13.6 vs 7.3 days per year; *P* < 0.001) and women (11.6 vs 7.8 days; *P* < 0.001) with lower income. Low income was associated with longer LOS in men (IRR 1.98; 95% CI, 1.54–2.56; *P* < 0.001) and women (IRR 1.42; 95% CI, 1.20–1.67; *P* < 0.001) after adjustment. Greater risks of increased LOS due to low income were also obtained from the analyses of inpatient services users only. Participants with LTC needs certification had less frequent physician visits and longer LOS than those without (eTable 4). Among participants without LTC needs certification, those with lower income had less frequent physician visits in men and longer LOS in men and women. Among participants with LTC needs certification, those with lower income had less frequent physician visits in men and women and longer LOS in men.

**Table 2.** Annual medical services utilization by gender and household income category

Indicators		Men		Women	
	
		Low income (*N* = 1,704)	Middle-to-high income (*N* = 11,148)	Low income (*N* = 7,483)	Middle-to-high income (*N* = 10,537)
***Physician visits, days***	Mean (SD)	14.1 (8.8)	16.1 (9.0)	14.8 (9.6)	16.1 (9.2)
	Median (IQR)	13 (9–18)	15 (10–21)	14 (8–20)	15 (10–21)

***Length of hospital stay, days***	Mean (SD)	13.6 (57.2)	7.3 (36.6)	11.6 (50.7)	7.8 (41.0)
*– Users only*^a^	*N* (%)	328 (19)	1,963 (18)	1,311 (18)	1,517 (14)
	Mean (SD)	70.6 (114.0)	41.4 (78.7)	66.1 (105.3)	54.4 (95.6)
	Median (IQR)	18 (6–72)	13 (4–35)	21 (7–61)	15 (6–44)

IQR, interquartile range; SD, standard deviation.

^a^Participants who had at least one claim submitted for inpatient services, *excluding* some surgical procedures with episode-based bundled payment, during the 12-month study period.

**Table 3.** Associations of low income with medical services utilization by gender

	Men				Women			
	
	Unadjusted		Adjusted^a^		Unadjusted		Adjusted^a^	
			
	IRR (95% CI)	*P* value	IRR (95% CI)	*P* value	IRR (95% CI)	*P* value	IRR (95% CI)	*P* value
***Physician visits***	0.87 (0.85–0.90)	<0.001	0.90 (0.87–0.92)	<0.001	0.91 (0.89–0.93)	<0.001	0.97 (0.95–0.99)	0.001

***Length of hospital stay***	1.86 (1.43–2.43)	<0.001	1.98 (1.54–2.56)	<0.001	1.48 (1.25–1.75)	<0.001	1.42 (1.20–1.67)	<0.001
*– Users only*^b^	1.71 (1.46–2.00)	<0.001	1.72 (1.48–2.00)	<0.001	1.21 (1.10–1.34)	<0.001	1.20 (1.09–1.32)	<0.001

CI, confidence interval; IRR, incidence rate ratio.

Negative binomial regression was used. IRRs were derived from exp(coefficient).

^a^Adjusted by 5-year age-group and LTC needs level.

^b^Participants who had at least one claim submitted for inpatient services, *excluding* some surgical procedures with episode-based bundled payment, during the 12-month study period.

### Healthcare expenditures

Descriptive statistics on annual healthcare expenditures are summarized in Table 4, and the relationship between low income and expenditures is presented in Table 5. Among the overall study participants (*N* = 30,872), a quarter of them (*n* = 7,895; 26%) used both medical and LTC services, and only a small proportion of the participants (*n* = 147; 0.5%) used LTC services only during the 12-month study period. Annual mean total expenditures were higher for both men (JPY 1,240,000 [USD 11,900] vs JPY 961,000 [USD 9,200]; *P* < 0.001) and women with lower income (JPY 1,457,000 [USD 14,000] vs JPY 1,002,000 [USD 9,600]; *P* < 0.001) compared with those with middle-to-high income. For total healthcare expenditures, medical expenditures accounted for a higher proportion, on average, for men (low income, 67%; and middle-to-high-income, 77%) compared to women (low income, 49%; and middle-to-high income, 63%). Men with lower income spent more on inpatient services (mean JPY 422,000 [USD 4,100] vs JPY 327,000 [USD 3,100]; *P* = 0.002) and had 20% (95% CI, 1–43%; *P* = 0.035) higher expenditures for inpatient services after adjustment. Similar results were observed for expenditures for inpatient services in women. Participants with lower income spent more on LTC services compared with those with middle-to-high income. The differences remained significant after adjusting for age group and LTC needs levels. For participants who used LTC services only, those with lower income spent around 10% more for institutional care services for men and women and home care services for men compared with those with middle-to-high income. Participants with LTC needs certification spent 3.6- to 4.5-fold higher total health expenditures than those without across gender and income (eTable 5). Participants with lower income tended to have higher total healthcare expenditures regardless of LTC needs certification. Higher total healthcare expenditures in participants with lower income mainly attributed to more spending on inpatient services among those without LTC needs certification. However, among participants with LTC needs certification, participants with lower income spent less on medical services but more on LTC services.

**Table 4.** Annual medical and long-term care expenditures (in JPY 1,000) by gender and household income category

Types of services				Men		Women	
	
				Low income (*N* = 1,704)	Middle-to-high income (*N* = 11,148)	Low income (*N* = 7,483)	Middle-to-high income (*N* = 10,537)
***Total: Medical and LTC services***			Mean (SD)	1,240 (1,715)	961 (1,433)	1,457 (1,705)	1,002 (1,411)
			Median (IQR)	461 (225–1,506)	402 (214–958)	567 (254–2,451)	408 (217–1,015)
	***Subtotal: Medical services***		Mean (SD)	826 (1,366)	738 (1,186)	718 (1,136)	636 (1,028)
			Median (IQR)	362 (191–767)	361 (201–717)	350 (199–685)	335 (190–610)
		***Inpatient services***	Mean (SD)	422 (1,250)	327 (1,055)	351 (1,079)	270 (957)
		*– Users only*^a^	*N* (%)	465 (27)	2,851 (26)	1,749 (23)	2,090 (27)
			Mean (SD)	1,548 (1,998)	1,278 (1,772)	1,502 (1,804)	1,363 (1,769)
			Median (IQR)	699 (276–1,865)	582 (224–1,551)	770 (317–1,936)	628 (296–1,636)
		***Outpatient services***	Mean (SD)	255 (450)	265 (446)	216 (325)	225 (341)
			Median (IQR)	141 (71–277)	161 (86–300)	138 (69–263)	153 (82–268)
		***Pharmaceuticals and pharmacy services by community pharmacy***	Mean (SD)	148 (200)	146 (180)	151 (161)	141 (151)
			Median (IQR)	103 (18–222)	107 (24–210)	119 (32–222)	108 (22–211)
	***Subtotal: LTC services***		Mean (SD)	414 (948)	223 (690)	740 (1,243)	366 (882)
		***Institutional care***	Mean (SD)	202 (770)	89 (503)	466 (1,129)	162 (688)
		*– Users only*^a^	*N* (%)	124 (7)	420 (4)	1,194 (16)	655 (6)
			Mean (SD)	2,780 (998)	2,352 (1,181)	2,918 (911)	2,599 (1,130)
			Median (IQR)	3,123 (2,471–3,429)	2,724 (1,324–3,283)	3,186 (2,613–3,514)	2,983 (1,780–3,477)
		***Home care***	Mean (SD)	212 (599)	135 (469)	274 (676)	204 (573)
		*– Users only*^a^	*N* (%)	352 (21)	1,700 (15)	2,245 (30)	2,375 (23)
			Mean (SD)	1,027 (949)	883 (883)	914 (969)	905 (907)
			Median (IQR)	739 (306–1,459)	561 (207–1,292)	554 (200–1,310)	574 (203–1,348)

IQR, interquartile range; LTC, long-term care; SD, standard deviation.

^a^Participants who had at least one claim submitted for inpatient services, *including* some surgical procedures with episode-based bundled payment, institutional care, or home care services, respectively, during the 12-month study period.

**Table 5.** Effect of low income on medical and long-term care expenditures by gender

			Men				Women			
	
			Unadjusted		Adjusted^a^		Unadjusted		Adjusted^a^	
			
			exp(β) (95% CI)	*P* value	exp(β) (95% CI)	*P* value	exp(β) (95% CI)	*P* value	exp(β) (95% CI)	*P* value
***Total: Medical and LTC services***			1.29 (1.20–1.39)	<0.001	1.09 (1.01–1.18)	0.024	1.45 (1.40–1.51)	<0.001	1.09 (1.05–1.14)	<0.001
	***Subtotal: Medical services***		1.12 (1.03–1.21)	0.007	1.05 (0.97–1.14)	0.223	1.13 (1.08–1.18)	<0.001	1.03 (0.99–1.08)	0.161
		***Inpatient services***	1.29 (1.10–1.52)	0.002	1.20 (1.01–1.43)	0.035	1.30 (1.18–1.44)	<0.001	1.16 (1.04–1.29)	0.007
		*– Users only*^b^	1.21 (1.06–1.39)	0.005	1.15 (1.00–1.31)	0.045	1.10 (1.02–1.19)	0.016	1.06 (0.98–1.15)	0.134
		***Outpatient services***	0.96 (0.88–1.05)	0.353	0.93 (0.85–1.01)	0.099	0.96 (0.92–1.00)	0.074	0.94 (0.90–0.98)	0.002
		***Pharmaceuticals and pharmacy services by community pharmacy***	1.02 (0.96–1.09)	0.542	1.00 (0.94–1.07)	0.936	1.07 (1.03–1.10)	<0.001	1.04 (1.01–1.07)	0.016
	***Subtotal: LTC services***		1.86 (1.59–2.16)	<0.001	2.39 (1.49–3.84)	<0.001	2.02 (1.90–2.16)	<0.001	1.64 (1.35–2.01)	<0.001
		***Institutional care***	2.28 (1.73–3.02)	<0.001	3.01 (0.93–9.68)	0.065	2.88 (2.59–3.21)	<0.001	2.75 (1.76–4.29)	<0.001
		*– Users only*^b^	1.18 (1.07–1.30)	0.001	1.13 (1.03–1.24)	0.012	1.12 (1.08–1.16)	<0.001	1.10 (1.07–1.14)	<0.001
		***Home care***	1.57 (1.32–1.87)	<0.001	2.10 (1.36–3.22)	0.001	1.34 (1.24–1.45)	<0.001	1.19 (0.98–1.46)	0.077
		*– Users only*^b^	1.16 (1.04–1.30)	0.009	1.11 (1.01–1.23)	0.031	1.01 (0.95–1.07)	0.751	1.03 (0.98–1.08)	0.241

CI, confidence interval; LTC, long-term care.

Generalized linear models with gamma distribution and log link function were used.

^a^Adjusted by 5-year age-group and LTC needs level.

^b^Participants who had at least one claim submitted for inpatient services, *including* some surgical procedures with episode-based bundled payment, institutional care, or home care services, respectively, during the 12-month study period.

## DISCUSSION

In this population-based study, we evaluated the disparities in health services utilization for people 75 or older under public insurance systems with universal coverage in Japan according to household income. Older people with lower income had less frequent contact with physicians and longer LOS, findings which were in line with previous studies on Japan in that people with low income had poorer access to outpatient care and higher hospitalization rates for both genders in the younger population below age 75.^19^^,^^20^ This study showed effect modifications by gender for the impact of lower income on medical services utilization and healthcare expenditures. Gender differences in health inequalities by socioeconomic status were also reported in previous studies.^10^^,^^13^^,^^22^^,^^23^ Several factors, including living status and psychosocial stresses due to lower income, can partly explain the effect modification observed in the present study.

The underlying reasons for inequalities observed in medical services utilization in older people should be considered. A possible mechanism is that older people with lower income might develop more severe illnesses, requiring inpatient care and a longer LOS, which might result from delayed or infrequent physician visits and suboptimal management of conditions that can be treated out of hospital.^24^ There is also the possibility that different types of inpatient services were used among income groups. As discussed by Penning and Zheng in the context of the Canadian health care system,^25^ individuals with lower income might rely more heavily on emergency hospitalization. Additionally, LOS might be prolonged due to social reasons; for example, for older men who have difficulty with housework by themselves. Further studied are needed to investigate types of inpatient services in more details among different income groups.

This study had several strengths. First, it included a wide range of individuals aged 75 years or older, irrespective of whether they lived or stayed in the community, LTC facilities, or hospitals, which enabled us to include data on more disabled people often excluded from studies on community-dwelling populations.^26^ Household income data collected for administrative purposes by the local government were almost complete (around 99% in this study), which is important since income is a sensitive question item in questionnaire surveys and different response rates among different income levels might introduce selection bias of the study population. Claims data are considered appropriate to evaluate the utilization of health services and healthcare expenditures.^27^ Participants could be followed on the database more exactly using both medical and LTC insurance data compared with a study with either of medical or LTC insurance data because insurance eligibility data for each participant were not available for this study as is the case with the National Claims Database.

However, this study is not without limitations. First, almost all individuals aged 75 years or older were retired and received pensions, meaning their living expenses depended more on assets or savings than for younger people. As such, for older people, wealth might be a more appropriate indicator of socioeconomic status than income.^11^^,^^28^^,^^29^ However, obtaining these data is practically difficult. Second, we could not exclude the possibility of residual confounding. Although LTC needs levels may be a good objective proxy of general health status, this was not always reflected by medical needs, given the high heterogeneity of older people. More than two-thirds of those aged 75 years or older remained independent. Longer-term data are needed to evaluate medical needs at the baseline with a sufficient follow-up period. Additionally, we did not consider non-financial barriers to healthcare access, such as geographical barriers.^18^ Third, people receiving public welfare were not included due to data unavailability. Fujino et al demonstrated a higher mortality risk in older men receiving public welfare, but the second lowest group, corresponding to the low-income group in our study, did not exhibit a higher risk of mortality.^22^ However, participants within the low-income category in our study had to pay out-of-pocket, unlike those on public welfare, which is why this population was considered for the evaluation of inequalities in healthcare access. Our study cohort also might be under-representative of people approaching their end-of-life by including only those who contributed to the database for at least 1 year, since medical and LTC services were used with different patterns in the last few months before death.^5^ Finally, since this study was based on a single city in Japan, generalizability might be limited for other areas in Japan or overseas.

In conclusion, this study suggests fewer physician consultations but increased use of inpatient services in older men and women with lower income in Japan. The household income categorization based on the LTC premiums in this study might be useful to determine inequality in terms of health services utilization. Improved access to healthcare, especially for outpatient services, for older people with lower income by providing some financial compensation may contribute to addressing health inequalities in older people in Japan. Further studies are needed to verify the utility of this categorization for health services research and epidemiological studies with various outcome measures as a potential proxy for socioeconomic status.

## APPENDIX A. SUPPLEMENTARY DATA

Figure S1. Flow diagram of study participants selection

Table S1. Baseline characteristics of study population with inpatient services use

Table S2. Baseline characteristics of study population with institutional care services use

Table S3. Baseline characteristics of study population with home care services use

Table S4. Annual medical services utilization by gender, household income category, and long-term care needs certification

Table S5. Annual medical and long-term care expenditures (in JPY 1,000) by gender and household income category
