# Supplementary material for: Household Income Relationship With Health Services Utilization and Healthcare Expenditures in People Aged 75 Years or Older in Japan: A Population-Based Study Using Medical and Long-term Care Insurance Claims Data
Source: J Epidemiol. 2019 Oct 5;29(10):377–83. doi: 10.2188/jea.JE20180055 (PMC6737189; doi:10.2188/jea.JE20180055)
Supplement: Supplementary file 1 [file je-29-377-s001.pdf]

eFigure 1. Flow diagram of study participants

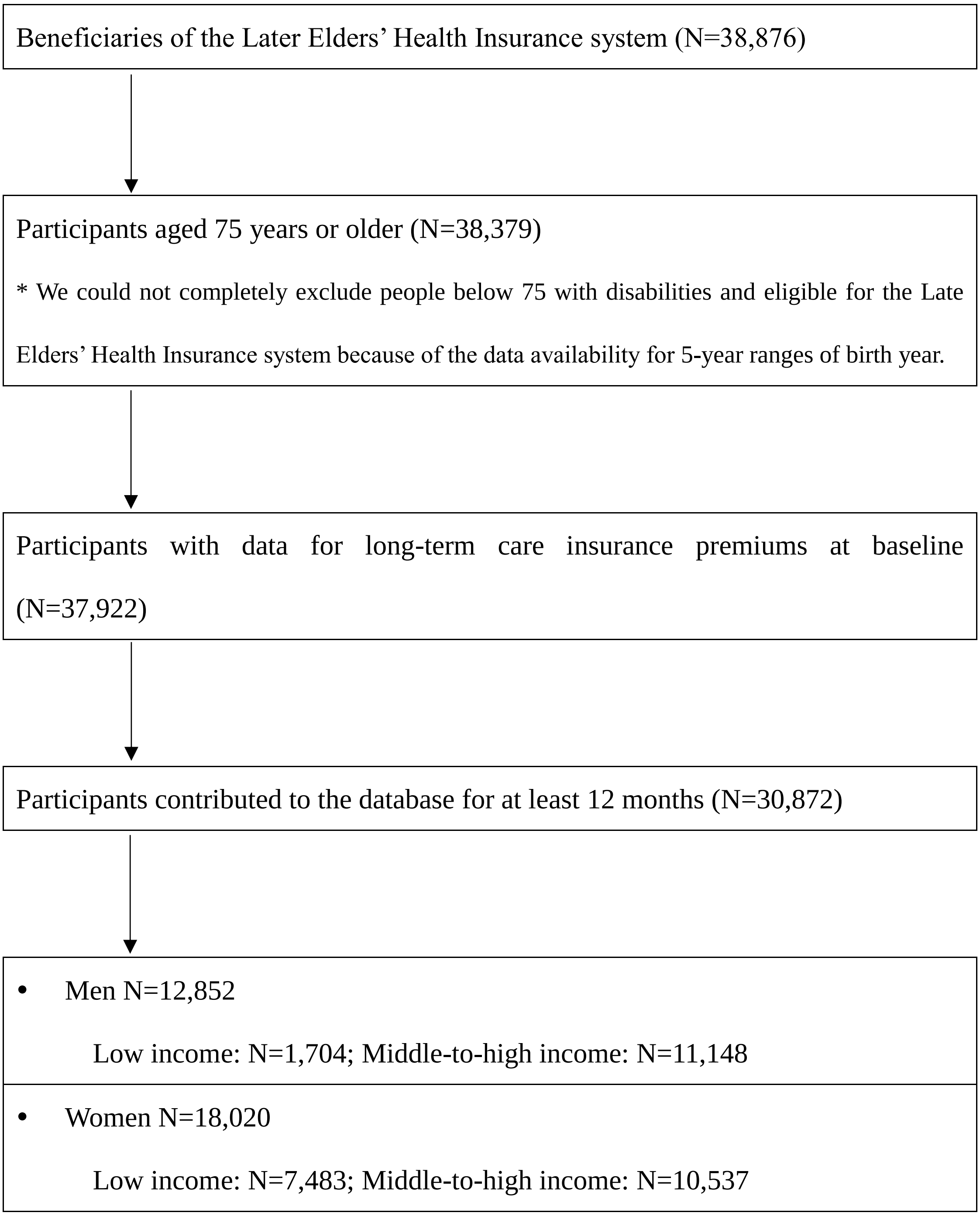

eTable 1. Baseline characteristics of study population with inpatient services use

		Men		Women	
		Low income (N=328)	Middle-to-high income (N=1,963)	Low income (N=1,311)	Middle-to-high income (N=1,517)
Age group, years	72–76[a]	94 (28.7)	535 (27.3)	183 (14.0)	336 (22.2)
	77–81	132 (40.2)	754 (38.4)	370 (28.2)	499 (32.9)
	82–86	72 (22.0)	442 (22.5)	347 (26.5)	380 (25.1)
	≥87	30 (9.2)	232 (11.8)	411 (31.4)	302 (19.9)
LTC needs level	Independent	181 (55.2)	1,352 (68.9)	514 (39.2)	876 (57.8)
	Support 1 & 2	26 (7.9)	117 (6.0)	128 (9.8)	114 (7.5)
	Care 1 & 2	48 (14.6)	234 (11.9)	296 (22.6)	258 (17.0)
	Care 3	33 (10.1)	108 (5.5)	116 (8.9)	96 (6.3)
	Care 4	22 (6.7)	85 (4.3)	109 (8.3)	72 (4.8)
	Care 5	18 (5.5)	67 (3.4)	148 (11.3)	101 (6.7)

LTC, long-term care. Figures are shown as frequencies (%).

Participants who had at least one claim submitted for inpatient services, *excluding* some surgical procedures with episode-based bundled payment, during the 12-month study period.

Some categories were combined because of the small numbers of participants in some cases.

[a] People <75 were only included if eligible for the Late Elders' Health Insurance system.

eTable 2. Baseline characteristics of study population with institutional care services use

		Men		Women	
		Low income (N=124)	Middle-to-high income (N=420)	Low income (N=1,194)	Middle-to-high income (N=655)
Age group, years	72–76[a]	31 (25.0)	48 (11.4)	74 (6.2)	60 (9.2)
	77–81	35 (28.2)	104 (24.8)	178 (14.9)	126 (19.2)
	82–86	34 (27.4)	136 (32.4)	318 (26.6)	193 (29.5)
	≥87	24 (19.4)	132 (31.4)	624 (52.3)	276 (42.1)
LTC needs level	Independent & Support 1 & 2[b]	11 (8.9)	61 (14.5)	98 (8.2)	74 (11.3)
	Care 1 & 2	30 (24.2)	128 (30.5)	360 (30.2)	205 (31.3)
	Care 3	37 (29.8)	107 (25.5)	269 (22.5)	165 (25.2)
	Care 4	33 (26.6)	78 (18.6)	233 (19.5)	105 (16.0)
	Care 5	13 (10.5)	46 (11.0)	234 (19.6)	106 (16.2)

LTC, long-term care. Figures are shown as frequencies (%).

Some categories were combined because of the small numbers of participants in some cases.

[a] People <75 years were only included if they were eligible for the Late Elders' Health Insurance system.

[b] This category includes people who were eligible for institutional care during the 12-month study period.

eTable 3. Baseline characteristics of study population with home care services use

		Men		Women	
		Low income (N=352)	Middle-to-high income (N=1,700)	Low income (N=2,245)	Middle-to-high income (N=2,375)
Age group, years	72–76[a]	103 (29.3)	284 (16.7)	214 (9.5)	296 (12.5)
	77–81	118 (33.5)	503 (29.6)	539 (24.0)	623 (26.2)
	82–86	77 (21.9)	508 (29.9)	696 (31.0)	718 (30.2)
	87–91	43 (12.2)	316 (18.6)	556 (24.8)	506 (21.3)
	≥92	11 (3.1)	89 (5.2)	240 (10.7)	232 (9.8)
LTC needs level	Independent[b]	52 (14.8)	309 (18.2)	260 (11.6)	346 (14.6)
	Support 1	27 (7.7)	177 (10.4)	276 (12.3)	254 (10.7)
	Support 2	31 (8.8)	168 (9.9)	307 (13.7)	254 (10.7)
	Care 1	66 (18.8)	328 (19.3)	491 (21.9)	531 (22.4)
	Care 2	88 (25.0)	350 (20.6)	419 (18.7)	468 (19.7)
	Care 3	42 (11.9)	213 (12.5)	241 (10.7)	276 (11.6)
	Care 4	27 (7.7)	119 (7.0)	142 (6.3)	150 (6.3)
	Care 5	19 (5.4)	36 (2.1)	109 (4.9)	96 (4.0)

LTC, long-term care. Figures are shown as frequencies (%).

[a] People <75 years were only included if they were eligible for the Late Elders' Health Insurance system.

[b] This category includes people who were eligible for home care during the 12-month study period.

eTable 4. Annual medical services utilization by gender, household income category, and long-term care needs certification

Indicators		Men		Women	
		Low income	Middle-to-high income	Low income	Middle-to-high income
**Participants without LTC needs certification**					
**N**		1,246	9,287	4,437	7,825
Physician visits, days	Mean (SD)	14.9 (8.3)	16.6 (8.8)	16.7 (8.9)	16.9 (8.8)
	Median (IQR)	13.5 (10–19)	15 (11–21)	15 (11–22)	15 (11–22)
Length of hospital stay, days	Mean (SD)	8.6 (46.3)	4.0 (24.6)	6.9 (41.3)	4.0 (27.7)
- Users only[a]	N (%)	181 (15)	1,352 (15)	514 (12)	876 (11)
	Mean (SD)	59.1 (108.8)	27.6 (59.3)	59.8 (107.6)	35.7 (75.6)
	Median (IQR)	12 (3–36)	9 (3–23)	13.5 (4–41)	10 (4–26.5)
**Participants with LTC needs certification**					
**N**		458	1,861	3,046	2,712
Physician visits, days	Mean (SD)	11.9 (9.7)	14.1 (9.8)	12.0 (9.9)	13.8 (10.0)
	Median (IQR)	11.5 (4–17)	13 (7–20)	11 (3–18)	13 (6–19)
Length of hospital stay, days	Mean (SD)	27.2 (78.0)	23.7 (68.5)	18.4 (61.5)	18.9 (64.4)
- Users only[a]	N (%)	147 (32)	611 (33)	797 (26)	641 (24)
	Mean (SD)	84.8 (118.9)	72.0 (104.1)	70.3 (104.1)	80.1 (112.5)
	Median (IQR)	29 (11–100)	28 (11–74)	27 (11–70)	28 (11–91)

IQR, interquartile range; LTC, long-term care; SD, standard deviation.

[a] Participants who had at least one claim submitted for inpatient services, *excluding* some surgical procedures with episode-based bundled payment, during the 12-month study period.

eTable 5. Annual medical and long-term care expenditures (in JPY 1,000) by gender and household income category

Types of services		Men: Low income	Men: Middle-to-high income	Women: Low income	Women: Middle-to-high income
**Participants without LTC needs certification**					
**N**		1,246	9,287	4,437	7,825
Total: Medical and LTC services	Mean (SD)	729 (1,319)	645 (1,032)	605 (1,006)	534 (830)
	Median (IQR)	321 (179–632)	334 (191–632)	307 (188–537)	306 (183–529)
Subtotal: Medical services	Mean (SD)	698 (1,251)	631 (1,006)	555 (914)	510 (782)
	Median (IQR)	318 (178–620)	332 (190–619)	300 (183–515)	303 (181–516)
Inpatient services	Mean (SD)	313 (1,098)	245 (861)	227 (866)	171 (692)
- Users only[a]	N (%)	280 (22)	2,099 (23)	752 (17)	1,269 (16)
	Mean (SD)	1,394 (1,967)	1,082 (1,542)	1,342 (1,714)	1,053 (1,423)
	Median (IQR)	566 (226–1,725)	463 (194–1,334)	585 (225–1,745)	492 (235–1,206)
Outpatient services	Mean (SD)	245 (429)	249 (410)	192 (217)	209 (298)
	Median (IQR)	141 (78–267)	159 (89–284)	141 (79–245)	152 (85–253)
Pharmaceuticals and pharmacy services by community pharmacy	Mean (SD)	139 (203)	137 (171)	135 (152)	130 (139)
	Median (IQR)	100 (20–206)	101 (23–200)	106 (30–196)	98 (22–194)

Subtotal: LTC services	Mean (SD)	32 (219)	15 (143)	50 (327)	24 (187)
**Participants with LTC needs certification**					
**N**		458	1,861	3,046	2,712
Total: Medical and LTC services	Mean (SD)	2,629 (1,891)	2,537 (2,003)	2,700 (1,753)	2,351 (1,816)
	Median (IQR)	2,295 (1,043–3,842)	2,041 (937–3,748)	2,803 (1,068–3,902)	1,894 (861–3,600)
Subtotal: Medical services	Mean (SD)	1,173 (1,590)	1,273 (1,743)	956 (1,367)	1,000 (1,472)
	Median (IQR)	579 (249–1,338)	641 (319–1,395)	486 (238–1,020)	487 (239–978)
Inpatient services	Mean (SD)	719 (1,555)	738 (1,665)	532 (1,313)	557 (1,437)
- Users only[a]	N (%)	185 (40)	752 (40)	997 (33)	821 (30)
	Mean (SD)	1,780 (2,026)	1,826 (2,208)	1,626 (1,868)	1,841 (2,113)
	Median (IQR)	911 (428–2,500)	922 (390–2,395)	904 (393–2,062)	958 (408–2,439)
Outpatient services	Mean (SD)	281 (503)	347 (584)	250 (435)	269 (440)
	Median (IQR)	141 (46–371)	180 (74–410)	132 (51–307)	158 (66–331)
Pharmaceuticals and pharmacy services by community pharmacy	Mean (SD)	173 (189)	187 (214)	173 (170)	173 (179)
	Median (IQR)	136 (6–273)	151 (25–275)	143 (37–260)	134 (19–262)
Subtotal: LTC services	Mean (SD)	1,455 (1,316)	1,264 (1,203)	1,745 (1,392)	1,351 (1,271)
	Median (IQR)	1,042 (272–2,645)	873 (220–2,185)	1,522 (382–3,151)	948 (224–2,364)
Institutional care	Mean (SD)	723 (1,331)	507 (1,121)	1,107 (1,528)	606 (1,235)

	- *Users only*[a]	N (%)	116 (25)	386 (21)	1,142 (37)	618 (23)
		Mean (SD)	2,854 (953)	2,446 (1,145)	2,954 (879)	2,657 (1,112)
		Median (IQR)	3,151 (2,581–3,435)	2,793 (1,662–3,338)	3,199 (2,664–3,522)	3,045 (1,972–3,490)
	Home care	Mean (SD)	732 (951)	757 (899)	637 (923)	746 (913)
	- *Users only*[a]	N (%)	300 (66)	1,391 (75)	1,985 (65)	2,029 (75)
		Mean (SD)	1,118 (975)	1,012 (907)	978 (987)	997 (930)
		Median (IQR)	857 (361–1,580)	728 (301–1,489)	632 (238–1,398)	689 (264–1,507)

IQR, interquartile range; LTC, long-term care; SD, standard deviation.

[a] Participants who had at least one claim submitted for inpatient services, *including* some surgical procedures with episode-based bundled payment, institutional care, or home care services, respectively, during the 12-month study period.
